# Exosomal PDL1 Suppresses the Anticancer Activity of CD8^+^ T Cells in Hepatocellular Carcinoma

**DOI:** 10.1155/2024/1608582

**Published:** 2024-10-09

**Authors:** Qi Hu, Shuai Chen, Rilin Deng, Hongyu Deng, Mingjing Peng, Xiaohong Wang, Shun Deng, Jinfeng Wang, Biaoming Xu, Yan Xu, Haizhen Zhu, Jinhai Zheng, Man Xia, Chaohui Zuo

**Affiliations:** ^1^Graduate Collaborative Training Base of Hunan Cancer Hospital, Hengyang Medical School, University of South China, Hengyang 421001, Hunan, China; ^2^School of Integrated Chinese and Western Medicine, Hunan University of Chinese Medicine, Changsha 410208, Hunan, China; ^3^Hunan Normal University School of Medicine, Changsha 410013, Hunan, China; ^4^Department of Gastroduodenal and Pancreatic Surgery, Translational Medicine Joint Research Center of Liver Cancer, Laboratory of Digestive Oncology, Hunan Cancer Hospital and The Affiliated Cancer Hospital of Xiangya School of Medicine, Clinical Research Center for Tumor of Pancreaticobiliary Duodenal Junction in Hunan Provincial, Central South University, Changsha 410013, Hunan, China; ^5^Key Laboratory of Tropical Translational Medicine of Ministry of Education, Department of Pathogen Biology, School of Basic Medicine and Life Science, Department of Clinical Laboratory of the Second Affiliated Hospital, The University of Hong Kong Joint Laboratory of Tropical Infectious Diseases, The Second Affiliated Hospital of Hainan Medical University, Hainan Medical University, Haikou 571199, Hainan, China; ^6^School of Biomedical Sciences, Hunan University, Changsha 410082, Hunan, China; ^7^Department of Gynecological Oncology, Hunan Cancer Hospital and The Affiliated Cancer Hospital of Xiangya School Medicine, Central South University, Changsha 410013, Hunan, China

## Abstract

Tumor microenvironment (TME) is essential for the development and progression of hepatocellular carcinoma (HCC). Exosomes participate in constructing TME by passing biological information, but the regulatory effect of PDL1 in exosomes on anticancer activity of CD8^+^ T cells in HCC still needs to be further explored. In this study, high level of PDL1 was found in plasma exosomes of HCC patients, which turned out to be significantly associated with the increased number of tumor nodules, the upregulated level of serum AFP, the raised tendency of TNM stage, and the poor prognosis of HCC. The expression of CD8 may be inhibited in HCC that is characterized with high level of PDL1, and the protein level of exosomal PDL1 was determined by intracellular PDL1 abundance. High level of exosomal PDL1 inhibited the proliferation and activation of CD8^+^ T cells, but exhibited limited effect on the proliferation of hepatic cancer cells. Moreover, the growth of tumors formed by hepatic cancer cells Hepa1–6 in C57L mice was significantly promoted by the exosomal PDL1, which might be caused by the inhibitory effect of exosomal PDL1 on CD8^+^ T cells. Thus, exosomal PDL1 promotes the development and progression of HCC through inhibiting the anticancer activity of CD8^+^ T cells. This study provides sights for understanding the oncogenic role of PDL1 and a reasonable explanation for the low efficacy of anti-PD1/PDL1 immunotherapies in HCC.

## 1. Introduction

Primary liver cancer (PLC) is the sixth most common cancer and the third mortality of cancer-related death in the world [1, 2]. Hepatocellular carcinoma (HCC) accounts for 75%–85% of PLC and develops in the context of chronic liver disease [3]. The main reason for the high mortality of HCC is the difficulty in early diagnosis and the lack of suitable and effective therapies for advanced patients [4–6], making the 5-year overall survival of HCC only 18% [7]. Immunotherapy has become a new focus in cancer therapy [8], and PD1/PDL1 signaling is a behand mechanism for immune evasion of HCC [9]. However, the heterogeneity of HCC and the associated immune microenvironment greatly lower the therapeutic efficacy of immune checkpoint blockade (ICB).

Exosomes are small vesicles secreted into the extracellular environment after the fusion of eukaryotic intracellular multivesicles and cell membrane, which are surrounded by lipid bilayer membrane with a diameter ranging from 40 to 160 nm [10]. Exosomes can be released by most types of cells represented by dendritic cells, platelets, and tumor cells and existed in body fluids represented by plasma, urine, and ascites [11–13]. The biological functions of exosomes are deeply dependent on their bioactive substances, such as lipids, metabolites, proteins, and nucleic acids [14]. Cancer-derived exosomes promote the formation of tumor microenvironment (TME), contributing to the development and progression of malignancies [15–17]. Moreover, exosomes are functioned as prognostic and therapeutic targets of cancer and carriers of anticancer drugs [18, 19].

TME, that constructed by the surrounded immune and inflammatory cells, cancer-related fibroblasts, extracellular matrix, growth factors, proteolytic enzymes, nearby microvessels, and stroma of cancer cells, supports the development and progression of malignancies [20, 21]. Immune microenvironment dominated by immune cells and nonimmune microenvironment dominated by fibroblasts together make up the TME [22], influencing multiple aspects of HCC such as energy metabolism regulation, stimulation signal supply, and inhibition signal avoidance [7, 23]. Immune microenvironment is formed by the complex interactions between cancer cells and host immunity, and the collapse of immune surveillance may lead to malignant transformation and poor prognosis of HCC [24]. Cancer-derived exosomes participate in regulating the immunosuppression and immune tolerance, involving in the formation of immune environment for HCC [25].

PDL1, also known as CD274 or B7-H1, is a 290 amino acid type I transmembrane protein encoded by CD274 gene [26]. In the normal condition, the activation of PD1/PDL1 signaling pathway is associated with the induction and maintenance of peripheral tolerance and the maintenance of T cell immune homeostasis, avoiding T cell overactivation and preventing immune-mediated tissue damage [27, 28]. In disease state, the ligand PDL1 generally interacts with the receptor PD1 to transmit negative feedback signal for controlling cellular immune response mediated by T and NK cells [29, 30]. Notably, the activation of PD1/PDL1 signaling may arrest T cell cycle in G1 phase, resulting in immune evasion of cancer cells [31]. Interestingly, the upregulated levels of PD1 and PDL1 are tightly correlated with the malignant transformation and poor prognosis of HCC [32].

Exosomes secreted by cancer cells may carry bioactive surface PDL1, inhibiting immune response represented by T cells [33–35]. For example, PDL1 on surface of exosomes released by glioblastoma stem cells inhibits the activation and proliferation of CD4^+^ and CD8^+^ T cells [36, 37]. In the model of malignant oral/esophageal injury induced by 4-nitroquine 1-oxide, PDL1 in exosomes that isolated from the supernatants of cultured mouth or head and neck squamous cell carcinoma cells prevents the infiltration of CD4^+^ and CD8^+^ T cells [38]. However, the regulatory role of exosomal PDL1 in HCC still needs to be further explored. Through conducting *in vitro* and *in vivo* experiments, this study mainly explored the regulatory effect and mechanism of exosomal PDL1 on HCC, which may provide insights for recognizing and improving the therapeutic efficacy of ICBs in cancer.

## 2. Materials and Methods

### 2.1. Clinical Specimens

Preoperative peripheral blood and postoperative tumor tissues were collected from 45 patients who underwent hepatectomy for HCC from January 2019 to December 2021 at the Department of Gastroduodenal and Pancreatic Surgery, Hunan Cancer Hospital. Prior to the operation in which samples were collected, HCC patients had not received surgery, chemotherapy, radiotherapy, and immunotherapy. Peripheral blood samples of healthy individuals were collected from 30 volunteers in Hunan Cancer Hospital. Protocols used for clinical specimens were approved by the Ethics Committee of Hunan Cancer Hospital, consisting with the Declaration of Helsinki. The collection of peripheral blood and postoperative tumor tissues was exerted with informed consent from HCC patients and volunteers.

### 2.2. ELISA Assay

ELISA plates (96-well) (Biolegend) that coated with Human PDL1 antibody (DY156, R&D SYSTEMS, Minneapolis, MN, USA), Human TNF*α* ELISA Kit (KIT10602, SinoBiology, Beijing, China), Mouse TNF*α* Matched ELISA Antibody Pair Set (SEKA50349, SinoBiology), Human IFN gamma ELISA Kit (KIT11725A, SinoBiology), and Mouse IFN gamma ELISA Kit (KIT50709, SinoBiology) were used for detecting protein levels of PDL1, TFN*α*, and IFN-*γ* in samples according to manufactures' instructions.

### 2.3. Cell Culture

The human hepatic cell line Huh7 (RRID: CVCL_0336) was obtained from the JCRB Cell Bank (Tokyo, Japan), the mouse hepatic cancer cell line Hepa1−6 (RRID: CVCL_0327) and the human embryonic kidney fibroblast line HEK293T (RRID: CVCL_0063) were obtained from ATCC (Manassas, VA, USA). Human-derived CD8^+^ T cells were isolated from the peripheral blood of healthy individuals. Huh7, Hepa1–6, and HEK293T cells were cultivated in DMEM medium (Thermo Fisher Scientific, Waltham, MA, USA) supplemented with 10% (*v*/*v*) FBS (Thermo Fisher Scientific) and penicillin–streptomycin (Thermo Fisher Scientific). The isolated CD8^+^ T cells were cultivated in RPMI-1640 medium (Thermo Fisher Scientific) supplemented with 10% (*v*/*v*) FBS and penicillin–streptomycin.

### 2.4. Isolation and Characterization of Exosomes

Exosomes derived from plasma of HCC patients and supernatants of cultured cells were isolated using ExoQuick-LP kit (System Biosciences, California, USA) and ExoQuick-TC kit (System Biosciences), respectively. Protein concentration of exosomes was determined using a BCA Protein Assay kit (Thermo Fisher Scientific, Waltham, USA). The size distribution of exosomes was measured by nanoparticle tracking analysis (NTA), and morphology of exosomes was observed under a JEM1400 transmission electron microscope (TEM) (Hitachi, Tokyo, Japan). Protein markers of exosomes including Alix, TSG101, CD63, and CD9 were analyzed by western blot.

### 2.5. Plasmid Construction

Protocol used for plasmid construction was presented in our previous research [29]. In brief, cDNA used for constructing PDL1 plasmid (human) and Pdl1 plasmid (mouse) were synthesized using total cellular RNA derived from Huh7 and Hepa1–6 cells, respectively. The coding sequences of PDL1 (human) and Pdl1 (mouse) were obtained and amplified using a KOD-Plus-Neo kit (TOYOBO, Japan), which were further inserted into p3×Flag-CMV-14 vector (Sigma–Aldrich, USA). The specific primers used for constructing overexpression plasmids of genes are listed in Table 1. Gene silencing plasmids were constructed using pGreenPuro shRNA cloning and expression lentivector (SBI, Palo Alto, CA, USA), and targets for gene silencing are listed below: human PDL1 (CGA ATT ACT GTG AAA GTC AAT), mouse Pdl1 (CCG AAA TGA TAC ACA ATT CGA), and negative control (TAA GGT TAA GTC GCC CTCG). Plasmids were amplified in *E. coli* DH5*α* competent cells and sequenced at Sangon Biotech (Shanghai, China).

### 2.6. Package and Infection of Lentivirus

HEK293T cells in the logarithmic phase were seeded into 100 mm dishes at 60%–70% cell coverage. After cultivation for 24 h, 8.0 µg of gene silencing plasmids, 8.0 µg of lentiviral packaging plasmid psPAX2 (Addgene, Watertown, MA, USA), and 2.7 µg of envelope plasmid pMD2.G (Addgene) were mixed into 300 µl of Opti-MEM serum-free medium. Meanwhile, 18 µl of Lipofectamine 2000 was mixed into 300 µl of Opti-MEM serum-free medium. After incubation at room temperature for 5 min, plasmids and Lipofectamine 2000 were mixed together and incubated at room temperature for 20 min, which were added into the culture medium of HEK293T cells. After transfection for 48 h, cell supernatants were collected, filtered with 0.45 µm filter membranes, and added into the medium of cultured cells (30%–40% coverage). To screen stably infected cells, puromycin (Thermo Fisher Scientific) was added to culture medium after viral infection for 72 h.

### 2.7. Quantitative Real-Time PCR (qRT-PCR)

Total RNA of cultured cells was extracted using TRIzol reagent (Thermo Fisher Scientific). Reverse transcription PCR of total RNA was performed using PrimeScript RT reagent Kit with gDNA Eraser (Takara, Kusatsu, Japan), and qRT-PCR was conducted using SYBR Premix Ex Taq II (Tli RNaseH Plus) (Takara). The specific primers used for qRT-PCR are listed in Table 2.

### 2.8. Western Blot

Cell or exosome lysates were obtained using RIPA lysis buffer (Thermo Fisher Scientific) and quantified by BCA method. Total protein was run on PAGE gels and then were electrotransferred onto PVDF membranes (Merck Millipore). Then, PVDF membranes were blocked with 5% skim milk and sequentially incubated with the primary and the second antibodies. Following antibodies were used for western blot according to the manufacturers' instructions: rabbit anti-CD9 (clone D3H4P, #13,403, CST, Danvers, MA, USA), rabbit anti-CD63 (clone E1W3T, #52,090 CST), Rabbit anti-TSG101 (102286-T38, Sino Biological, Beijing, China), rabbit anti-PDL1 (clone E1L3N, #13,684, CST), and mouse anti-GAPDH (clone 6C5, MAB374, Merck Millipore, Darmstadt, Germany). Protein bands on PVDF membranes were detected using the Chemiluminescent Substrate System (Thermo Fisher Scientific), and GAPDH was used as the internal control.

### 2.9. Isolation and Treatment of CD8^+^ T Cells

Peripheral blood mononuclear cells (PBMCs) were isolated from blood samples of a total of five healthy individuals using Ficoll-PaqueTM PLUS (GE Health, Uppsala, Sweden), and CD8^+^ T cells were purified from PBMCs by magnetic cell sorting using CD8^+^ T Cell Isolation Kit (Miltenyi Biotec, Bergisch Gladbach, Germany). Purified CD8^+^ T cells were cultured using RPMI-1640 medium in six-well cell culture plates, which were incubated with 2.0 μg of exosomes at different times. Proliferation of CD8^+^ T cells was examined by CCK8 assay.

### 2.10. CCK-8 Assay

Huh7 cells (2000 cells per well in a 96-well plate) in 100 μl of culture medium were seeded in per well of a 96-well plate, and purified CD8^+^ T cells (20,000 cells per well in a 96-well plate) in 100 μl of culture medium were stimulated with 2 μg/ml anti-CD3 (BioLegend, USA) and 2 μg/ml anti-CD28 (BioLegend) antibodies for 24 h. After cell cultivation at 37°C in a humidified incubator with 5% CO_2_ for 12 h, exosomes were added to the culture medium. To determine the proliferation of cells, 10 μl of 1 mg/ml CCK-8 solution (Promega) was added into the culture medium and incubated at 37°C in a humidified incubator with 5% CO_2_ for 3 h. Then, the optical density (OD) value at 450 nm was measured using a microplate reader.

### 2.11. Immunohistochemistry (IHC)

The paraffin-embedded tissues were sliced, dewaxed, hydrated, and immersed in 3% H_2_O_2_. Next, sections were subjected to antigen retrieval by boiling in a sodium citrate solution (0.01 M and pH = 6.0), which were blocked with normal goat serum (C-0005, Shanghai Haoran Bio Technologies Co., Ltd., Shanghai, China) for 20 min. Sections were incubated with primary antibody at 4°C overnight, treated with the peroxidase horseradish-labeled secondary antibody at room temperature for 2 h, and followed by DAB staining for 5 min at room temperature. Sections were further counterstained by hematoxylin, blued with ammonia water, and dehydrated with gradient ethanol, cleared by xylene, sealed by neutral balsam, and observed under a microscope. Following antibodies were commercially obtained and used for IHC staining: rabbit anti-PDL1 (clone E1L3N, #13,684, CST), rabbit anti-CD8*α* (clone, D8A8Y, #85,336, CST), rabbit anti-CD8*α* (clone D4W2Z, #98,941, CST), rabbit anti-PDL1 (clone D5V3B, #64,988, CST), and goat antirabbit IgG (HRP-linked) (AP132P, Merck Millipore, Darmstadt, Germany).

Protein levels of stained sections were evaluated based on the extent of IHC staining (the percentages of positive cells were scored: 0, 0%–10% staining; 1, 11%–25% staining; 2, 26%–50% staining; 3, 51%–75% staining; 4, 76%–100% staining) and the intensity of IHC staining (0, no staining; 1, weak staining; 2, moderate staining; and 3, strong staining). A total score of more than 3 was defined as positive expression of PDL1 proteins [39]. CD8^+^ T cells that infiltrated into tumor tissues were evaluated according to the previous report [40]. Images presented at high-power field (×400) were captured for analysis, which was counted using Image J software.

### 2.12. Animal Experiment

C57L mice (male, 6-week-old) were obtained from Hunan SJA (Changsha, China). In total, 5 × 10^6^ Hepa1−6 cells were administrated into each C57L mouse by subcutaneous injection. Mice were randomly assigned to each group, and tumor volumes were monitored on the indicated days. After cell injection for 10 days, mice were treated with 50 μg of exosomes with different levels of PDL1 in 150 μl of sterile PBS by tail vein injection every 3 days for a total of four times. Mice were sacrificed on day 30 postcell injection, and tumors were collected, weighed, and staining by IHC. Protocols used for animal experiments was performed and approved by the Institutional Animal Care and Use Committee of Hunan Cancer Hospital. All applicable international, national, and/or institutional guidelines for the care and use of animals were followed in the study.

### 2.13. Statistical Analysis

Excel 2021 (Microsoft Corporation, Redmond, WA, USA) was used to analyze statistical data, and GraphPad Prism 8 (GraphPad Software, San Diego, CA, USA) was used to draw statistical images. Chi-square analysis (two-sided) was performed to determine the correlation between the exosomal PDL1 level and clinicopathological features. Kaplan–Meier analysis (log-rank test) was carried out to determine the correlation between the exosomal PDL1 level and overall survival. The high and low levels of exosomal PDL1 were determined according to the median value. The significance of difference between two groups was analyzed by Student's two-sided *t*-test and is represented as mean ± SD. Significant differences were considered at  ^*∗*^*p* < 0.05,  ^*∗∗*^*p* < 0.01,  ^*∗∗∗*^*p* < 0.001, and not significant (ns).

## 3. Results

### 3.1. High Level of Exosomal PDL1 Protein May Predict Malignant Transformation and Poor Prognosis of HCC

**Figure 1:**
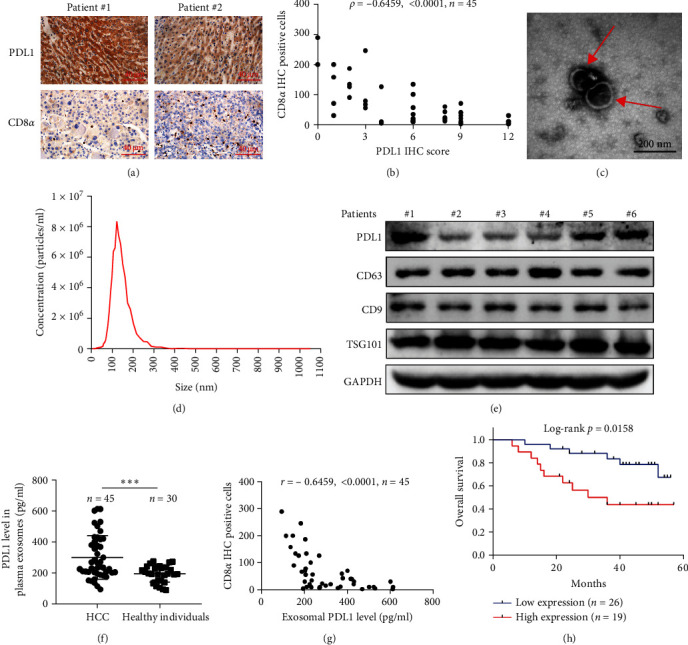
Expression profile and regulatory role of PDL1 in plasma exosomes of HCC. (A) IHC staining showing the protein levels of PDL1 and CD8*α* in representative cancer tissues of HCC. Scale bar 40 μm. (B) Statistical analysis of IHC staining with PDL1 or CD8*α* antibody in tissue sections of HCC, which was evaluated by Spearman's correlation (*ρ*). (C and D) TEM showing the morphology (C) and NTA showing the size (D) of isolated exosomes derived from plasma of HCC patients. Scale bar in (C) is 200 nm. (E) Western blot for protein levels of PDL1 and exosomal markers including CD63, CD9, and TSG101. (F) Scatter plots showing the expression profiles of exosomal PDL1 protein in plasma of HCC patients and healthy individuals, as examined by ELISA. Data were analyzed by Student's two-sided *t*-test and are represented as mean ± SD. ^∗∗∗^*p*<0.001. (G) Statistical analysis of PDL1 level examined by IHC staining or CD8*α* level examined by ELISA of HCC, which was evaluated by Pearson's correlation (*r*). (H) Kaplan–Meier analysis showing the influence of exosomal PDL1 on 5-year overall survival of HCC, as determined by log-rank test. The high and low level of exosomal PDL1 level was determined according to the median value.

The progression and poor prognosis of HCC may be caused by the collapse of immune monitoring function. Here, we explored the regulatory role of PDL1 in immune microenvironment of HCC. It turned out that the protein level of CD8*α* determined by IHC staining was lower in cancer tissues that were characterized with higher expression of PDL1 protein, and vice versa (Figure 1A). Notably, a negative Spearman's correlation (*ρ* = −0.6459, *p* < 0.0001) was calculated between the protein levels of PDL1 and CD8*α* in 45 HCC specimens (Figure 1B). Thus, PDL1 level in cancer tissues is negatively associated with the number of infiltrated CD8^+^ T cells. Interestingly, increasing evidence suggested that exosomes secreted by cancer cells participate in regulating the immune microenvironment and tolerance [41, 42]. Therefore, we speculated that exosomal PDL1 derived from hepatic cancer cells might participate in regulating the anticancer activity of CD8^+^ T cells in HCC. To validate our speculation, peripheral blood of 45 HCC patients and 30 healthy individuals were collected to isolate the plasma exosomes and quantify the protein level of exosomal PDL1. The morphology of plasma exosomes observed by TEM was vesicular (Figure 1C), and the diameter of plasma exosomes determined by NTA was mainly ranged from 40 to 160 nm (Figure 1D). Meanwhile, the specific protein markers of exosomes including CD63, CD9, and TSG101 were examined by western blot (Figure 1E). All these findings indicated the successful isolation of plasma exosomes.

Notably, the high level of PDL1 protein in plasma exosomes of some HCC patients was observed by western blot (Figure 1E), indicating the potential regulation of exosomal PDL1 on immune microenvironment of HCC. Through conducting ELISA analysis, the protein level of PDL1 in plasma exosomes of HCC patients was found to be significantly higher than that in healthy individuals (Figure 1F). Meanwhile, the association between pathological characteristics of HCC patients and protein level of exosomal PDL1 was analyzed by conducting Chi-square analysis (Table 3). Although the protein level of exosomal PDL1 was not significantly associated with sex, age, T stage, N stage, and M stage, high level of exosomal PDL1 was significantly associated with the increased number of tumor nodules, plasma AFP level, and total TNM stage (Table 3). A negative Pearson's correlation (*r*) was obtained between the protein levels of exosomal PDL1 in plasma examined by ELISA and CD8*α* examined by IHC staining (Figure 1G). Thus, high level of exosomal PDL1 may promote the development and progression of HCC. Moreover, the Kaplan–Meier analysis showed that high level of exosomal PDL1 was significantly associated with the poor prognosis of HCC, and low level of exosomal PDL1 brought a nearly 30% advantage on 5-year overall survival rate of HCC (Figure 1H). These data suggested that high level of exosomal PDL1 may predict malignant transformation and poor prognosis of HCC.

### 3.2. Exosomal PDL1 Inhibits the Proliferation and Activation of CD8^+^ T Cells

**Figure 2:**
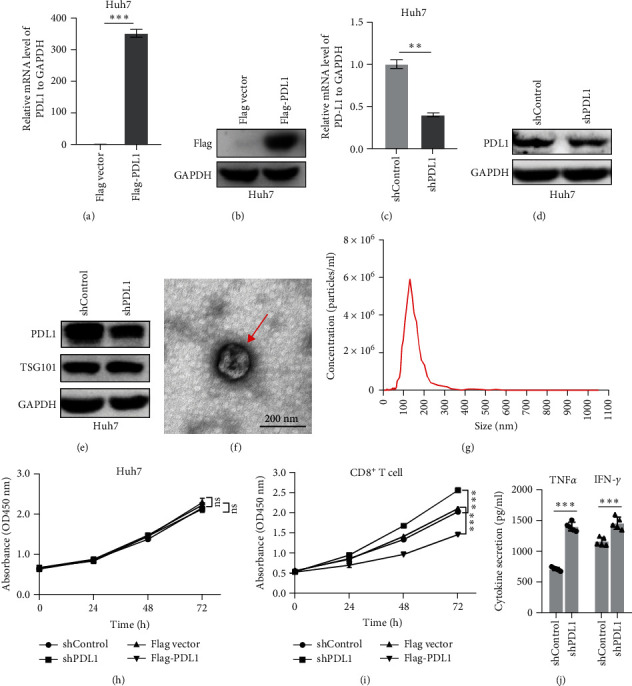
Regulatory effect of exosomal PDL1 on proliferation of CD8^+^ T and hepatic cancer cells. (A–D): qRT-PCR (A and C) and western blot (B and D) for PDL1 level in Huh7 cells with the overexpression or knockdown of PDL1. (E) Western blot for exosomal PDL1 level in supernatants of PDL1-silenced Huh7 cells. (F and G): TEM (F) and NTA (G) for isolated exosomes derived from the supernatants of PDL1-silenced Huh7 cells. (H) Proliferation of Huh7 cells with the overexpression or silencing of PDL1, as assayed by CCK8. (I) Proliferation of CD8^+^ T cells with the treatments of exosomes derived from the supernatants of Huh7 cells, as assayed by CCK8. PDL1 was overexpressed or silenced in Huh7 cells. (J) ELISA for TNF*α* and IFN-*γ* levels in supernatants of human CD8^+^ T cells after treatments with exosomes. Data were analyzed by Student's two-sided *t*-test and are represented as mean ± SD. ^∗∗^*p*<0.01, ^∗∗∗^*p*<0.001.

To explore the regulatory effect of exosomal PDL1 derived from hepatic cancer cells on CD8^+^ T cell activity, we stably overexpressed PDL1 or silenced PDL1 in Huh7 cells (Figure 2A–D). Interestingly, the protein level of PDL1 in exosomes derived from the supernatants of cultured Huh7 cells was downregulated with the knockdown of cellular PDL1 level (Figure 2E). The examined TSG101 protein by western blot, the observed morphology by TEM, and the calculated diameters by NTA suggested the successful isolation of exosomes from the supernatants of Huh7 cells (Figure 2E–G). Notably, neither overexpressing nor silencing PDL1 influenced the proliferation activity of Huh7 cells (Figure 2H), but the proliferation of CD8^+^ T cells was significantly inhibited by exosomes derived from PDL1-overexpressed Huh7 cells and promoted by exosomes derived from PDL1-silenced Huh7 cells (Figure 2I). Meanwhile, the protein levels of TNF*α* and IFN-*γ* that secreted by CD8^+^ T cells were significantly upregulated after treatment with exosomes derived from PDL1-silenced Huh7 cells (Figure 2J). Thus, exosomal PDL1 may inhibit the proliferation and activation of CD8^+^ T cells in HCC.

### 3.3. Exosomal PDL1 Promotes Tumor Growth In Vivo

**Figure 3:**
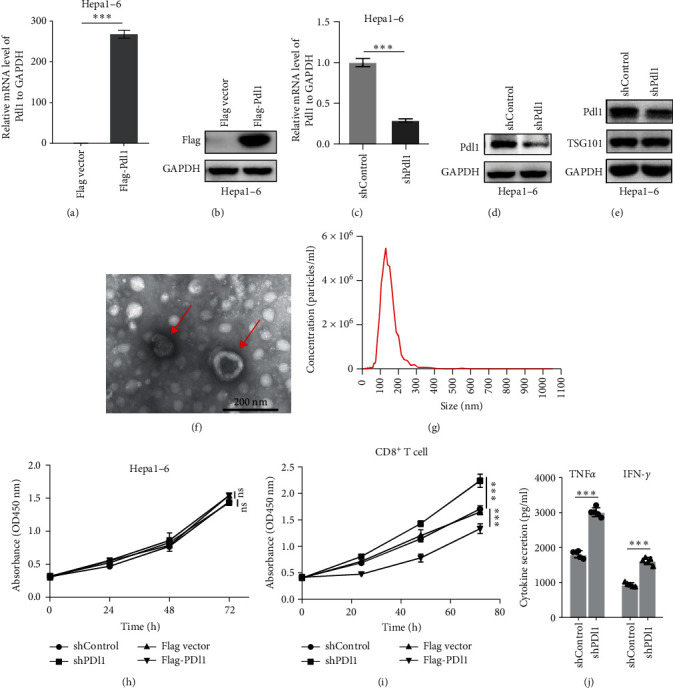
Isolation of exosomes derived from the supernatants of cultured Hepa1–6 cells with different levels of PDL1. (A–D): qRT-PCR (A and C) and western blot (B and D) for PDL1 level in Hepa1–6 cells with the overexpression or knockdown of PDL1. (E) Western blot for protein level of PDL1, TSG101, and GAPDH in exosomes derived from the supernatants of PDL1-silenced Hepa1–6 cells. (F and G) TEM and NTA analysis for the isolated exosomes derived from the supernatants of Pdl1-silenced Hepa1–6 cells. (H) Proliferation of Hepa1–6 cells with the overexpression or silencing of Pdl1, as assayed by CCK8. (I) Proliferation of CD8^+^ T cells after treatments of exosomes derived from the supernatants of Hepa1–6 cells, as assayed by CCK8. Pdl1 was overexpressed or silenced in Hepa1–6 cells. (J) ELISA for TNF*α* and IFN-*γ* levels in supernatants of mice CD8^+^ T cells after treatments with exosomes. Data were analyzed by Student's two-sided *t*-test and are represented as mean ± SD. ^∗∗∗^*p*<0.001.

Next, mouse-derived Pdl1 was also overexpressed by plasmid transfection or silenced by lentiviral infection in Hepa1−6 cells (Figure 3A–D). Similar to that of human-derived hepatic cancer cells Huh7, silencing Pdl1 in mouse-derived hepatic cancer cells Hepa1−6 cells downregulated the protein level of PDL1 in exosomes (Figure 3E–G). Interestingly, exosomes with different levels of Pdl1 protein had no influence on the proliferation of Hepa1−6 cells (Figure 3H), which was highly consistent with the finding in Huh7 cells. Meanwhile, the proliferation and cytokine secretions (TNF*α* and IFN-*γ*) of mice CD8^+^ T cells were significantly upregulated after treatment with exosomes derived from Pdl1-silenced Hepa1−6 cells (Figure 3I,J). These findings approved the inhibitory effect of exosomal PDL1 on proliferation and activation of CD8^+^ T cells.

**Figure 4:**
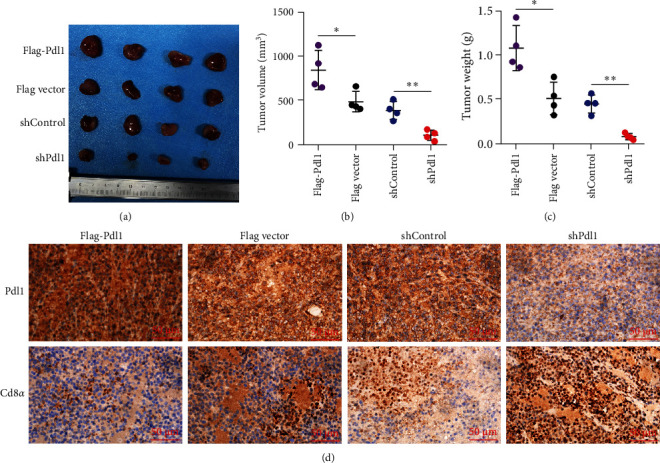
Influence of exosomal Pdl1 on growth of tumors formed by Hepa1–6 cells. (A) The formed tumors by Hepa1–6 cells in C57L mice. After subcutaneous injection of Hepa1–6 cells in mice for 10 days, exosomes derived from the supernatants of Hepa1–6 cells that stably transfected with Flag-Pdl1 plasmid or Flag vector or stably infected with shControl or shPdl1 lentivirus were administrated into mice by tail vein injection every 3 days for four consecutive injections. The smallest scale of the ruler, 1 mm. (B and C) Statistical analysis for tumor volume and tumor weight in (A). (D) Representative images of IHC staining for protein levels of Pdl1 and CD8 in mouse tumor tissues. Scale bar 50 μm. Data were analyzed by Student's two-sided *t*-test and are represented as mean ± SD.  ^*∗*^*p* < 0.05,  ^*∗∗*^*p* < 0.01.

Exosomes may enter the blood and lymphatic system and spread throughout the body, affecting the growth of distant tumors. Herein, C57L mice were applied to evaluate the regulatory effect of exosomal PDL1 on tumor growth formed by Hepa1−6 cells. After tumor formation by Hepa1−6 cells for 10 days, exosomes derived from Pdl1-overexpressed or -silenced Hepa1−6 cells were administrated into mice by tail vein injection, respectively. It turned out that exosomes derived from Pdl1-overexpressed Hepa1−6 cells promoted the tumor growth in mice, whereas exosomes derived from Pdl1-silenced Hepa1−6 cells inhibited tumor growth *in vivo* (Figure 4A–C). The positive role of exosomal Pdl1 in promoting tumor growth suggested that exosomal PDL1 may participate in constructing the immunosuppressive microenvironment of HCC. Moreover, the results of IHC staining showed that the protein level of Pdl1 was significantly higher while that of Cd8*α* was significantly lower in tumor tissues of Pdl1-overexpred group, and vice versa (Figure 4D and Table 4). Thus, exosomal PDL1 may promote the exhaustion of CD8^+^ T cells in HCC.

## 4. Discussion

Emergence of ICBs has reformed the therapeutic methods of malignancies. Several ICB monotherapies, such as anti-PD1 and anti-PDL1, have been approved for therapying the advanced HCC [43, 44]. However, the therapeutic efficacy of ICBs in HCC is limited by cancer heterogeneity and immune microenvironment, and the objective remission rate of ICB immunotherapy for advanced HCC is only 16%–20% at present [45, 46]. Analyzing the immune microenvironment of HCC may provide insights for exploring the behand mechanism of immune evasion, which may help to develop effective strategies for diagnosis and therapy of HCC. This study mainly explored the inhibitory role of exosomal PDL1 in immune microenvironment of HCC, providing a new sight into recognizing the immunosuppressive role of immune checkpoint PDL1.

ICB therapy has become a primary choice for multiple cancers, but the unsatisfactory clinical response at present reveals the necessity for exploring the behind mechanism. Interestingly, exosomal PDL1 is involved in immune regulation of malignant tumors, which may be responsible for the failure of ICB therapy [47–49]. Some studies have reported the regulatory role and mechanism of exosomal PDL1 in cancer progression. For example, exosomal PDL1 accelerates the progression of nonsmall cell lung cancer by inhibiting T cell activity or promoting T cell apoptosis [50]. The protein level of exosomal PDL1 derived from head and neck cancer cells is significantly associated with disease progression, number of tumor nodules, and tumor stage, which may downregulate CD69 antigen level of cytotoxic T cells and reduce CD8^+^ T cell activity [51]. In this study, we uncovered the high level of exosomal PDL1 protein in plasma exosomes of HCC patients. Moreover, the 5-year overall survival rate of HCC was decreased by the high level of exosomal PDL1, and high level of exosomal PDL1 was significantly associated with the increased number of tumor nodules, the upregulated level of serum AFP, and the raised tendency of TNM stage. Thus, exosomal PDL1 promotes the malignant transformation of HCC, functioning as a promising biomarker for clinical diagnosis and prognostic factor.

The connection between PDL1 and PD1 is a way for human body to create immune response homeostasis, but cancer cells manipulate PDL1 to escape the immune surveillance. PD1 on activated T cells, and its interaction with PDL1 contributes to T cell inhibition [52, 53]. Exosomal PDL1 protein has the same membrane topology with PDL1 protein on cell surface [54], indicating the similar biofunctions of exosomal PDL1 and cell surface PDL1. In this study, high level of PDL1 protein was found in peripheral blood of HCC patients. Meanwhile, the expression CD8 might be inhibited in HCC characterized with high expression of PDL1, and high level of exosomal PDL1 protein was found in plasma exosomes of HCC patients. Consequently, high level of exosomal PDL1 inhibited the proliferation and activation of CD8^+^ T cells, thus promoting the growth of tumors formed by HCC cells. On the contrary, decreasing exosomal PDL1 may reverse the inhibitory state of cancer cells on T cell activity, increasing the number of functional CD8^+^ T cells and inhibiting the growth of tumors formed by hepatic cancer cells. ICB therapy is becoming a promising choice for the treatment of a variety of cancers, but its sustainable response in clinic is still low. In the immune microenvironment of HCC, anti-PD1 antibody binds to PD1 on the surface of T cells and anti-PDL1 antibody binds to PDL1 on the surface of cancer cells, which will activate T cells and eliminate tumors. However, exosomal PDL1 may cause the therapeutic failure of PDL1 antibody, resulting in the exhaustion of T cells and the failure of anti-PD1/PDL1 therapy. Our findings in this study uncovered the high level of exosomal PDL1 in plasma of HCC and also revealed the inhibitory effect of exosomal PDL1 on anticancer activity of CD8^+^ T cells.

## 5. Conclusions

PDL1 protein is highly expressed in plasma exosomes of HCC patients, which may induce CD8 T cell exhaustion and predict the poor prognosis. Exosomal PDL1 may be a promising marker for diagnosis and therapy of HCC in future clinic. Although our study revealed that the exist of exosomal PDL1 may be a behand reason for the failure of ICB therapy, the detailed mechanism of exosomal PDL1 in immune microenvironment of HCC needs to be further explored. Since most HCC patients are diagnosed at advanced stages, the number of tissue samples used for this study is somewhat limited.

## Figures and Tables

**Table 1:** Primers used for plasmid construction.

Plasmids	Primers (5′−3′)
Flag-PDL1 (human)	Forward: GGAATTCATGAGGATATTTGCTGTCTTTATAT
	Reverse: GCTCTAGACGTCTCCTCCAAATGTGTATCACTT

Flag-Pdl1 (mouse)	Forward: GGAATTCATGAGGATATTTGCTGGCATTATAT
	Reverse: GCTCTAGACGTCTCCTCGAATTGTGTATCATTT

**Table 2:** Primers used for qRT-PCR.

Genes	Primers (5′−3′)
PDL1 (human)	Forward: TGTGACCAGCACACTGAGAAT
	Reverse: TCAGTGCTACACCAAGGCAT

Pdl1 (mouse)	Forward: GGCATTTGCTGAACGCAT
	Reverse: CAATTAGTGCAGCCAGGT

GAPDH (human)	Forward: AACGGATTTGGTCGTATTGGG
	Reverse: CCTGGAAGATGGTGATGGGAT

Gapdh (mouse)	Forward: AAGAGGGATGCTGCCCTTAC
	Reverse: TCTACGGGACGAGGAAACAC

**Table 3:** Clinicopathologic features of different levels of PDL1 protein in plasma exosomes of HCC patients.

Groups	Cases	Number of HCC patients		*p*
		High	Low	
Sex				
Male	30 (66.67%)	13 (68.42%)	17 (65.38%)	0.282
Female	15 (33.33%)	6 (31.58%)	9 (34.62%)	
Age				
<55	13 (28.89%)	8 (42.11%)	5 (19.23%)	0.721
≥55	32 (71.11%)	11 (57.89%)	21 (80.77%)	
Tumor nodules				
1	31 (68.89%)	7 (36.84%)	24 (92.31%)	0.017
≥2	14 (31.11%)	12 (63.16%)	2 (7.69%)	
Serum AFP				
> 400 ng/ml	29 (64.44%)	15 (78.95%)	14 (53.85%)	0.041
≤400 ng/ml	16 (35.56%)	4 (21.05%)	12 (57.69%)	
T stage				
T1	6 (13.33%)	3 (15.79%)	3 (11.54%)	0.058
T2	7 (15.56%)	3 (15.79%)	4 (15.38%)	
T3	23 (51.11%)	10 (52.63%)	13 (50.00%)	
T4	9 (20.00%)	3 (15.79%)	6 (23.08%)	
N stage				
N0	21 (46.67%)	10 (52.63%)	11 (42.31%)	0.143
N1	13 (28.89%)	4 (21.05%)	19 (73.08%)	
N2	11 (24.44%)	5 (26.32%)	6 (23.08%)	
M stage				
M0	25 (55.56%)	10 (52.63%)	15 (57.69%)	0.122
M1	20 (44.44%)	9 (47.37%)	11 (42.31%)	
TNM stage				
I	8 (17.78%)	2 (10.53%)	6 (23.08%)	0.034
II	10 (22.22%)	3 (15.79%)	7 (26.92%)	
III	21 (46.67%)	11 (57.89%)	10 (38.46%)	
IV	6 (13.33%)	3 (15.79%)	3 (11.54%)	

**Table 4:** Statistical analyses for protein levels of Pdl1 and CD8a in mice tumors examined by IHC staining.

Groups	Sample numbers	Staining score (mean ± SD)		*p*	
		Pdl1	Cd8a	Pdl1	Cd8a
Flag-Pdl1	4	5.50 ± 0.58	1.75 ± 0.50	0.020	0.040
Flag vector	4	3.75 ± 0.96	3.00 ± 0.82		
shControl	4	4.00 ± 0.82	3.50 ± 1.29	0.032	0.012
shPdl1	4	2.00 ± 0.82	5.25 ± 0.96		

## Data Availability

The datasets generated and/or analyzed during the current study are available from the corresponding author upon reasonable request.
